# Feasibility of longitudinal prostate R_2_^⁎^ measurements in patients undergoing stereotactic ablative radiotherapy on an MRI-linac

**DOI:** 10.1016/j.phro.2026.101103

**Published:** 2026-10-06

**Authors:** Chris Moore, Asher Ezekiel, Vodathi Bamunuarachchi, Ereny Saad, Anubhav Datta, Peter Hoskin, Ananya Choudhury, Marcel van Herk, David L. Buckley, Michael J. Dubec, Damien J. McHugh

**Affiliations:** a CMPE, The Christie NHS Foundation Trust, Manchester, UK; b Division of Cancer Sciences, University of Manchester, Manchester, UK; c Clinical Oncology, The Christie NHS Foundation Trust, Manchester, UK; d Clinical Radiology, The Christie NHS Foundation Trust, Manchester, UK; e Clinical Oncology, Mount Vernon Cancer Centre, Northwood, UK; f Biomedical Imaging, University of Leeds, Leeds, UK

**Keywords:** MRI-linac, SABR, Prostate Cancer, Imaging-Biomarkers, R_2_*, Hypoxia

## Abstract

**Background and Purpose:**

Tissue R_2_* (1/T_2_*), sensitive to deoxyhaemoglobin, is a proposed hypoxia biomarker in prostate cancer. This exploratory work investigates prostate R_2_* and volume throughout and after hypo-fractionated radiotherapy and the effects of androgen deprivation therapy (ADT).

**Materials and methods:**

Fifteen patients (seven with ADT) received five-fraction stereotactic-ablative-radiotherapy (SABR) on a 1.5 T Elekta Unity MRI-linac, with R_2_* mapping at MR-Simulation and treatment visits 1, 3, 5. Six patients had follow-up imaging post-treatment.

Treatment-induced whole-prostate and tumour R_2_*/volume differences were investigated using repeated-measures analysis-of-variance.

ADT effects on baseline whole-prostate R_2_*/volume were investigated using unpaired *t*-tests. Spearman correlations between whole-prostate R_2_* and volume were calculated before and during treatment.

**Results:**

Whole-prostate R_2_* (mean difference [95% CIs] = −1.77 [−2.17 to −0.498] s^−1^, *p* = 0.003) and tumour R_2_* (−3.11 [−5.78 to −1.44] s^−1^, *p* = 0.003) decreased, whole-prostate volume (8.05 [4.76 to 11.7] cm^3^) increased after two fractions.

Follow-up whole-prostate R_2_* (*p* = 0.62) and volume (*p* = 0.16) were not significantly different from baseline.

ADT patients had higher baseline whole-prostate R_2_* (*p* = 0.006) and lower volume (*p* = 0.003). ADT did not impact treatment-induced changes (*p* = 0.51).

Baseline whole-prostate R_2_*/volume were negatively correlated (*r* = −0.75, *p* = 0.002); treatment-induced R_2_*/volume changes were not correlated *r* = 0.44, *p* = 0.11).

**Conclusions:**

Treatment-induced whole-prostate and tumour R_2_* changes were measured after two SABR fractions. Post-treatment whole-prostate R_2_* and volume were not significantly different from baseline.

Further work is required to confirm the mechanism(s) causing observed treatment effects.

## Highlights

• Whole-prostate and tumour R_2_* were measured on an MRI-Linac during radiotherapy.
• Whole-prostate and tumour R_2_* decreased, prostate volume increased after two fractions
• Androgen Deprivation Therapy strongly affects baseline R2* and volume.
• Follow up whole prostate R2* and volume were not significantly different from baseline.
• Radiation-induced hyperaemia and oedema are suggested as mechanisms for changes.

## Graphical abstract

**Figure:**
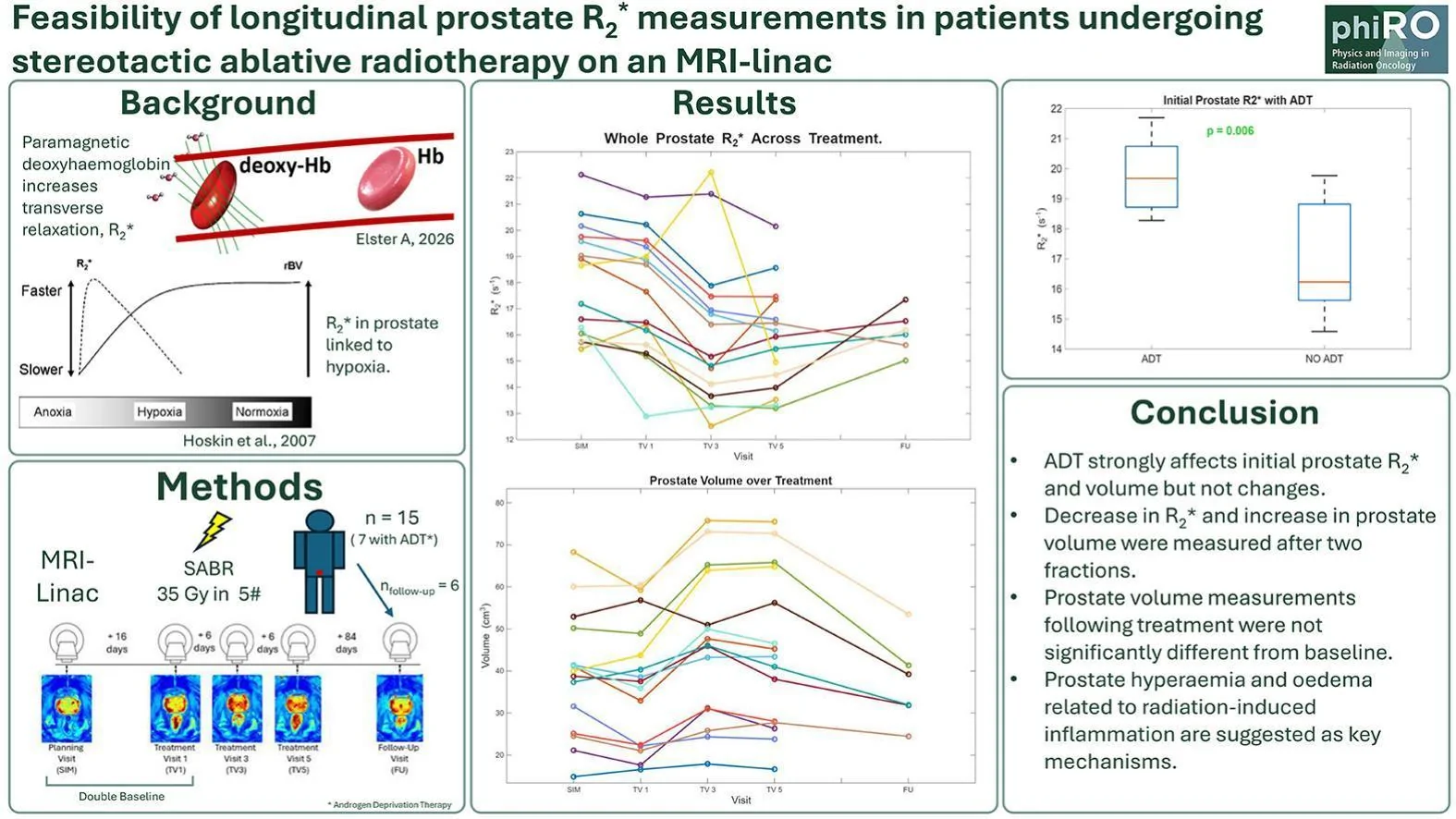

## 1 Introduction and Motivation

Prostate cancer is one of the most common cancers in men [1], with radiotherapy treating up to 50% of patients [2]. Prostate cancer hypoxia is of particular interest for radiotherapy, as increased hypoxia is associated with treatment resistance and poorer outcomes [3]. However, prostate cancer hypoxia is not routinely quantified clinically due to lack of robust, practical biomarkers [4]. Imaging biomarkers are of interest as they permit non-invasive assessment of biological properties of healthy and cancerous tissue which could aid tumour delineation, enable prediction of treatment response, and allow monitoring and intervention in treatments [5]. Hypoxia imaging biomarkers in prostate cancer could permit treatment intensification or de-intensification to improve local control or reduce treatment-related side effects [6].

Hybrid MRI-radiotherapy (MRI-linac) systems permit daily imaging of tumour biology, so provide the opportunity to monitor tissue hypoxia changes during treatment, potentially guiding adaptive treatment [7].

One candidate hypoxia biomarker is R_2_^⁎^, the relaxation rate associated with decay of transverse magnetisation depending on intrinsic spin-spin interactions and local field inhomogeneities; R_2_* is the inverse of T_2_*, the effective transverse relaxation time. R_2_* is sensitive to the presence of deoxyhaemoglobin, which is paramagnetic so increases R_2_^⁎^. This is commonly referred to as the blood‑oxygen level dependent (BOLD) phenomenon [8]. R_2_* has been shown to have high sensitivity but low specificity for identifying prostate cancer hypoxia [9], [10].

Conventional prostate cancer external-beam radiotherapy treatment (EBRT) is delivered over approximately 20 fractions [11]. However, there is a move towards hypo-fractionated treatments as improved outcomes are associated with higher doses per fraction [11], [12]. MRI-linacs offer the potential to deliver these higher doses while sparing healthy tissue. Furthermore, reducing the number of patient visits is more economical, improves patient experience, and is more environmentally sustainable [11], [13], [14], [15]. As this treatment modality is taken-up, it is important to investigate biomarkers in this context.

Whole-prostate R_2_^⁎^ measurements, made by Bano et al. on an MRI-linac throughout conventional radiotherapy, suggested a decrease in R_2_* after treatment [16]; but no measurements of prostate R_2_^⁎^ during hypo-fractionated treatment have been reported. It is possible that the increased dose per fraction could have a different effect on R_2_* compared to conventional treatment.

In this exploratory feasibility study, the primary aim was to assess whether R_2_^⁎^ changes in the prostate could be measured during hypo-fractionated stereotactic ablative radiotherapy (SABR) treatment [17] on an MRI-Linac. A secondary aim was to attempt to interpret the R_2_* results, so prostate volume measurements were assessed. Additionally, androgen deprivation therapy (ADT), which is routinely offered in prostate cancer treatments, has been shown to affect prostate blood flow, and therefore R_2_* [18], [19]. As such, ADT effects on prostate R_2_* and volume changes, were also investigated.

## 2 Methods and materials

### 2.1 Patient recruitment

Patients with intermediate-risk prostate cancer undergoing radical hypo-fractionated treatment of 35 Gy in five fractions were recruited to the MOMENTUM trial (NCT04075305) to receive their treatment on a 1.5 T MRI-linac (Elekta Unity) system. Patients were offered ADT prior to radiotherapy, according to standard of care [19]. Patients were invited separately to participate in the MR-BIO study (NCT04903236), to receive follow-up imaging approximately three months after their course of radiotherapy.

### 2.2 Image acquisition

All imaging was acquired on the MRI-Linac. Patients were imaged at their MR simulation (MR-SIM) scan and at treatment visits (TV) 1, 3 and 5, with the follow-up sub-cohort undergoing a further imaging session following the conclusion of treatment. Patients were imaged with a twelve-echo T_2_^⁎^ mapping sequence (Table 1; sequence exam card available upon request). At the treatment visits the T_2_^⁎^ mapping sequence was acquired before the therapy was delivered, and therefore the measurements from SIM and TV1 visits could be used as a double-baseline to assess repeatability. At all timepoints patients received an enema prior to treatment to reproducibly prepare their bowel for imaging and treatment [20].

**Table 1:** Summary of the parameters of the T2* mapping sequence.

Image Parameter	Value
Sequence Type	2D Steady-state spoiled gradient-echo (M_FFE)
Field of view [mm]	384 × 384
Slice Thickness/Spacing [mm]	5 / 5
Acquisition Matrix	256 × 256
Pixel Size (acquired/reconstructed) [mm]	1.2 × 1.2 / 1.2 × 1.2
Repetition Time [ms]	1379
Echo Times [ms]	4.6, 9.2, 13.8, 18.4, 23.0, 27.6, 32.2, 36.8, 41.4, 46.0, 50.6, 55.2
Bandwidth per pixel [Hz]	543
Flip Angle [^o^]	80
Parallel Imaging Type/Factor	SENSE / 2
Number of Averages	2
AI Reconstruction	No

### 2.3 Image analysis

T_2_^⁎^ maps were generated by fitting a mono-exponential decay curve to signal acquired from the individual echo images of the T_2_^⁎^ mapping sequence, against echo time (TE), on a voxel-wise basis, using a non-linear least-squares fit algorithm (Python, lmfit, v1.2.2). An R_2_^⁎^ map was generated from the T_2_^⁎^ map by calculating 1/T_2_^⁎^ for each voxel.

A clinical oncologist in urology contoured the patients' whole prostate and dominant intraprostatic lesion (DIL) at each visit. Whole-prostate contours were defined on the image from the T_2_^⁎^ mapping sequence with TE = 36.8 ms (selected because the balance between signal-to-noise ratio and contrast was determined to be suitable for prostate delineation) and transferred directly to the R_2_^⁎^ maps. The DILs were defined for each visit on the daily T_2_-weighted (T_2_w) image as they were not discernible on the T_2_* images. To aid localisation and delineation of the DIL, pre-treatment diagnostic multiparametric-prostate MR scans for patients, were displayed on a separate monitor [21]. The daily T_2_w image was rigidly registered to the T_2_^⁎^w image (as the contrast was like the T_2_w image) permitting transfer of DIL contours to the R_2_^⁎^ maps. Contouring and registration were carried out using Raystation v2025 (RaySearch Laboratories, Stockholm, Sweden).

Registration accuracy, particularly in small tumours at the edge of the prostate, and the presence and severity of susceptibility-related artefacts associated with bowel gas, were reviewed visually and qualitatively for DIL ROIs overlaid on each of the twelve-echo images used to obtain the T_2_* map. If the registration failed such that that a significant portion of the DIL was outside of the prostate, or bowel-gas artefact was so severe that it obscured a significant part of the DIL, then the DIL R_2_* for that visit was excluded from subsequent analysis.

Whole-prostate and DIL median R_2_^⁎^ were then extracted from the R_2_^⁎^ maps. Prostate and DIL contour volumes were also calculated.

### 2.4 Statistical analysis

Statistical analysis was performed in MATLAB R2025a (Mathworks, Nantick MA) and Python 3 (Python Software Foundation).

### 2.5 Whole-prostate R_2_* and volume repeatability and changes throughout treatment

Whole-prostate R_2_* repeatability was assessed using the within-subject coefficient of variation (*wCV*), calculated from the double-baseline (SIM and pre-beam TV1) measurements [21]:

(i) $$\mathrm{wCV}\;\left(\%\right)=100\times \sqrt{\frac{\sum_{\mathrm{patients}\;}{\left(\frac{\Delta_{i}}{m_{i}}\right)}^{2}}{2n_{p}}\;}$$

where *Δ*_*i*_ and *m*_*i*_ are the difference and mean of the baseline measurements for a patient *i* and n_p_ is the total number of patients.

Following Shapiro-Wilks normality tests and Maunchly's sphericity tests, repeated-measures analysis of variance (rm-ANOVA) was used to determine whether there was a significant change across the course of treatment for whole-prostate R_2_^⁎^, and if pre-treatment ADT was a significant co-factor for this change. Post-hoc analysis (with Bonferroni corrections to correct for multiple comparisons [22]) was performed to determine whether results at treatment visits were significantly different from baseline. This was the primary analysis which was performed to determine whether there were any R_2_* changes throughout treatment. Subsequent analysis was carried out to aid interpretation of results and should be considered exploratory.

The above repeatability and rm-ANOVA analysis was also carried out on whole-prostate ROI volume measurements.

### 2.6 DIL R_2_* and volume repeatability and changes throughout treatment

Image and registration quality assessment was carried out on each patient visit to determine whether the DIL result was suitable for inclusion in this analysis. Where a visit was assessed to be not suitable for analysis, all the DIL R_2_* and volume results for that patient were excluded from the subsequent analysis.

The repeatability and rm-ANOVA analysis outlined in the previous section was repeated for DIL R_2_* and DIL volume for the full four-visit cohort to determine whether these agreed with or differed from the whole-prostate results.

### 2.7 R_2_* and volume follow-up comparison to baseline

Paired *t*-tests were used in the follow-up sub-cohort to test for any significant difference between baseline and follow-up measurements.

Image and registration quality assessment was carried out on the baseline and follow-up results to determine whether the DIL results were suitable for analysis. Where either a baseline or follow-up result was determined to be unsuitable for analysis then that patient was excluded.

### 2.8 Effect of ADT on baseline whole-prostate R_2_* and volume

Unpaired two-tailed *t*-tests were used to compare whole-prostate R_2_^⁎^ and volume at baseline between patients who did and did not receive ADT before treatment to determine if there was any significant difference between the two groups.

### 2.9 Comparison of whole-prostate and DIL R_2_* at baseline and during treatment

Paired two-tailed *t*-tests were used to compare whole-prostate and DIL R_2_* at baseline and TV3 to determine if there was a significant difference before or during treatment.

### 2.10 Correlations between baseline R_2_* and volume and differences

Spearman correlation coefficients were calculated between whole-prostate R_2_* and prostate volume at baseline, and between differences from baseline to TV3 (the greatest mean difference), to determine if there was any relation between the measured changes.

## 3 Results

Fifteen patients (median age = 68; range 54 to 80), all with Gleason score 7, recruited to the study underwent the four-visit imaging protocol; seven of whom received ADT prior to radiotherapy. Six of the fifteen patients opted to have follow-up imaging after treatment (mean follow-up time = 81 days, range = 63 to 93 days). For the initial whole-prostate R_2_* and volume analysis, all fifteen patients were included each with measurements at all four treatment time points (SIM, TV1, TV3 & TV5).

Within-subject coefficient of variation was 4.4% for whole-prostate R_2_* (Fig. 1A) and 8.9% for whole-prostate volume (Fig. 1C).

**Fig. 1:**
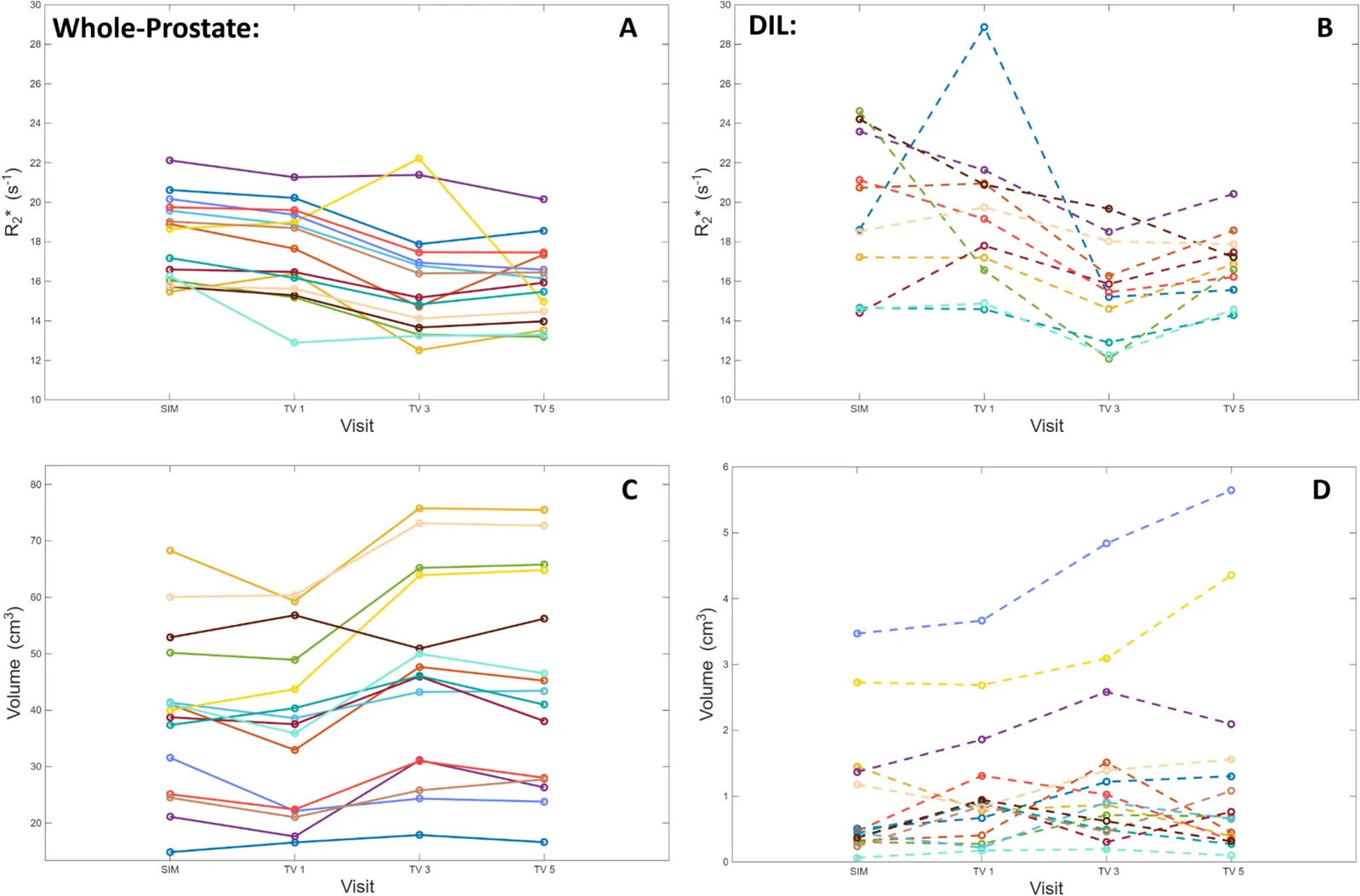
Plots of whole-prostate R_2_* (A) and volume (C) and DIL R_2_* (B) and volume (D) measurements throughout treatment. Each point represents the ROI median value for the R_2_* measurements, or the ROI volume for the volume measurements from the given visit. Each trace represents an individual patient, with colours corresponding to the same patient in each panel and those in Fig. 2.

Normality was observed for all visits for whole-prostate R_2_* and volume, but sphericity was violated, so the lower-bound correction was used. The rm-ANOVA analysis revealed significant changes across treatment for whole-prostate R_2_* (Fig. 1A) (*p* = 0.002) and volume (Fig. 1C) (*p* = 0.005). Whether the patient received ADT was not a significant co-factor (*p* = 0.51).

Post-hoc analysis revealed significant cohort-level whole-prostate mean R_2_* decreases from baseline to treatment visit 3 (*p* = 0.003, mean difference [95% Confidence Interval (CI)] = −1.77 [−2.17 to −0.498] s^−1^/−11.1 [−13.1 to −3.91] %) and treatment visit 5 (*p* = 0.001, mean difference = −1.98 [−2.57 to −1.64] s^−1^/−11.1 [−14.7 to −7.56] %); and significant cohort-level whole-prostate volume increases from baseline to treatment visit 3 (*p* = 0.001, mean difference [95% CI] = 8.05 [4.76 to 11.7] cm^3^/22.1 [14.3 to 33.2] %) and treatment visit 5 (*p* = 0.006, mean difference [95% CI] = 6.70 [4.97 to 12.0] cm^3^/17.1 [7.58 to 25.3] %). Four patients were excluded from subsequent DIL analysis due to image/registration quality assessment failures. Therefore, eleven patients were included in that analysis.

Within-subject coefficient of variation was 22.6% for DIL R_2_* (Fig. 1B) and 30.6% for DIL volume (Fig. 1D).

Normality was observed for DIL R_2_* and volume for all visits; sphericity was observed for DIL R_2_*, but violated for volume, so the lower-bound correction was used. The rm-ANOVA revealed a significant change across treatment for DIL R_2_* (*p* = 0.031), but no significant change for DIL volume (*p* = 0.18).

Post-hoc analysis revealed that for DIL R_2_* there were significant decreases from baseline to TV3 (*p* = 0.003, mean difference [95% CI] = −3.11 [−5.78 to −1.44] s^−1^/−14.6 [−24.1 to −4.03] %) and TV5 (*p* = 0.046, mean difference [95% CI] = −3.15 [−5.49 to −2.57] s^−1^/−14.4 [−24.3 to −11.5] %). For the six patient cohort who received follow-up imaging, the rm-ANOVAs detected significant changes across treatment in whole-prostate R_2_*(*p* = 0.03) and volume (*p* = 0.03), but post-hoc analysis showed that there was no significant difference between baseline and follow-up for whole-prostate R_2_^⁎^ (Fig. 2A) (*p* = 0.62, mean difference [95% CI] = −0.299 [−1.71 to 0.981] s-^1^/ -1.91 [−9.37 to 5.98]) or volume (Fig. 2B) (*p* = 0.16, mean difference [95% CI] = −6.83 [−11.4 to −2.59] cm^3^/ -16.5 [−23.4 to −4.35] %).

**Fig. 2:**
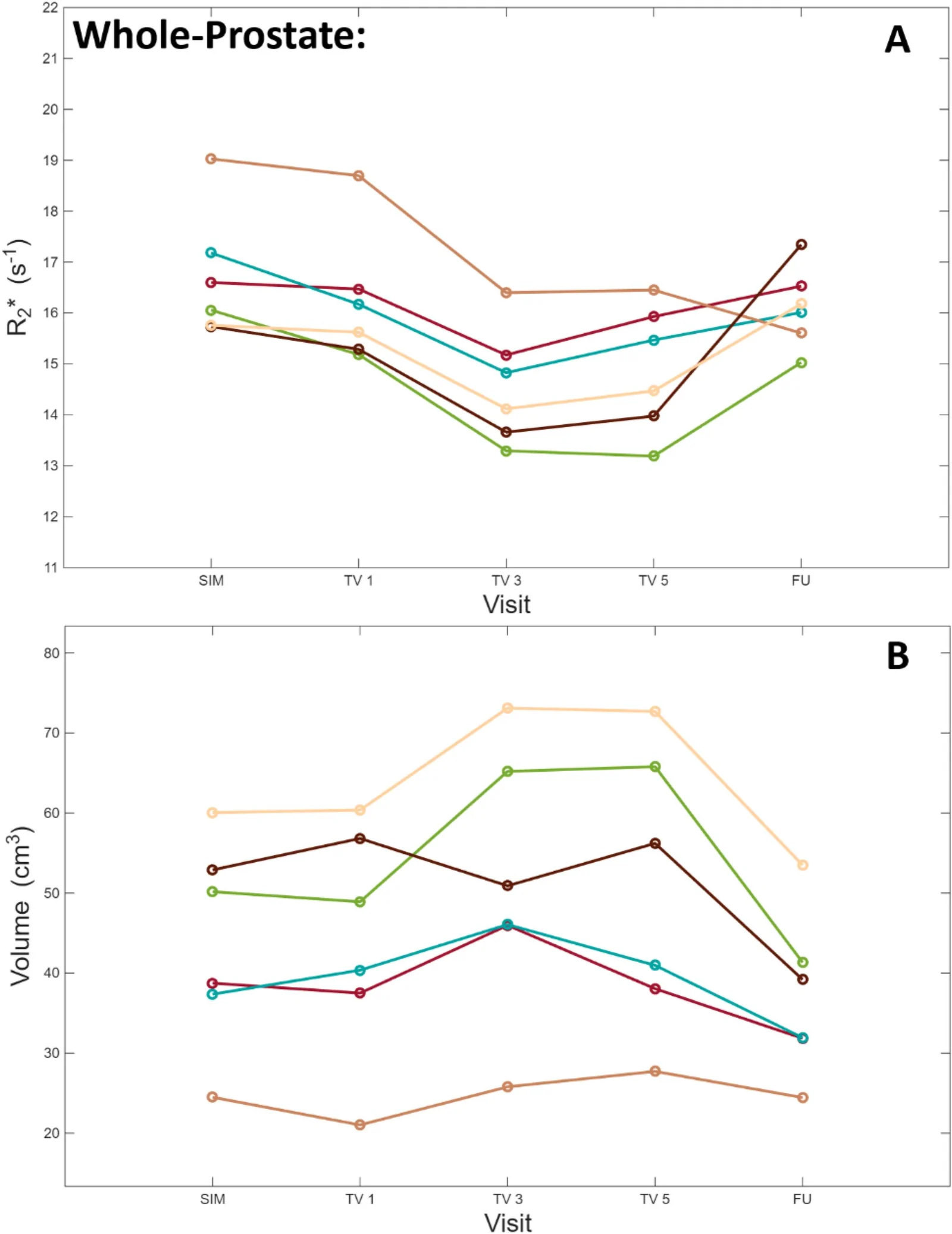
Plots of whole-prostate R_2_* (A) and volume (B) measurements throughout treatment and at follow-up. Each point represents the ROI median value for the R_2_* measurements, or the ROI volume for the volume measurements from the given visit. Each trace represents an individual patient, with colours corresponding to the same patient in each panel and those in Fig. 1.

Image/registration quality assessment excluded three patients from DIL analysis, so this was not performed due to insufficient data.

All patients were included in the ADT analysis. Therefore, there were 7 patients in the ADT group and 8 in the non-ADT group. At baseline, whole-prostate R_2_^⁎^ (Fig. 3A) was higher (*p* = 0.006) and volume (Fig. 3B) was lower (*p* = 0.003) for patients who received ADT.

**Fig. 3:**
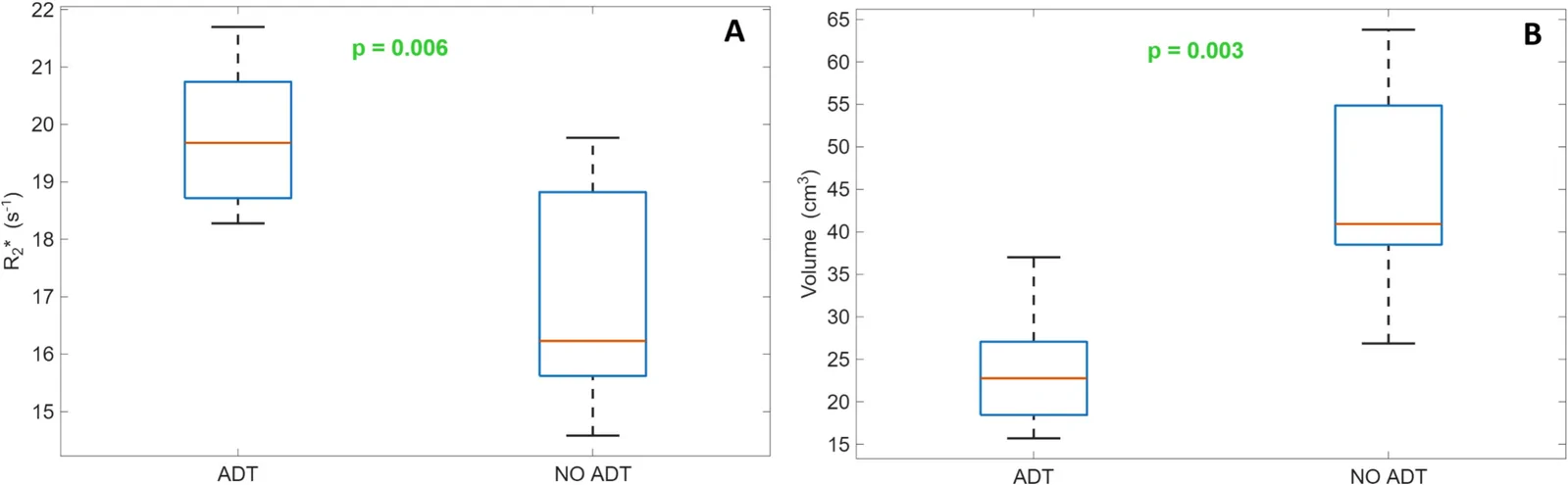
Comparison of whole-prostate median R_2_* (A) and volume (B) between patients who did and did not receive ADT.

There was no significant difference between whole-prostate and DIL R_2_* at baseline (Fig. 4A) (*p* = 0.063) or TV3 (Fig. 4B) (*p* = 0.40). There was a strong, statistically significant negative correlation between baseline whole-prostate R_2_* and volume (Fig. 5A) (*r* = − 0.75, *p* = 0.002). For whole-prostate R_2_* and volume changes between baseline and TV3, there was no significant correlation (Fig. 5B) (*r* = 0.44, *p* = 0.11).

**Fig. 4:**
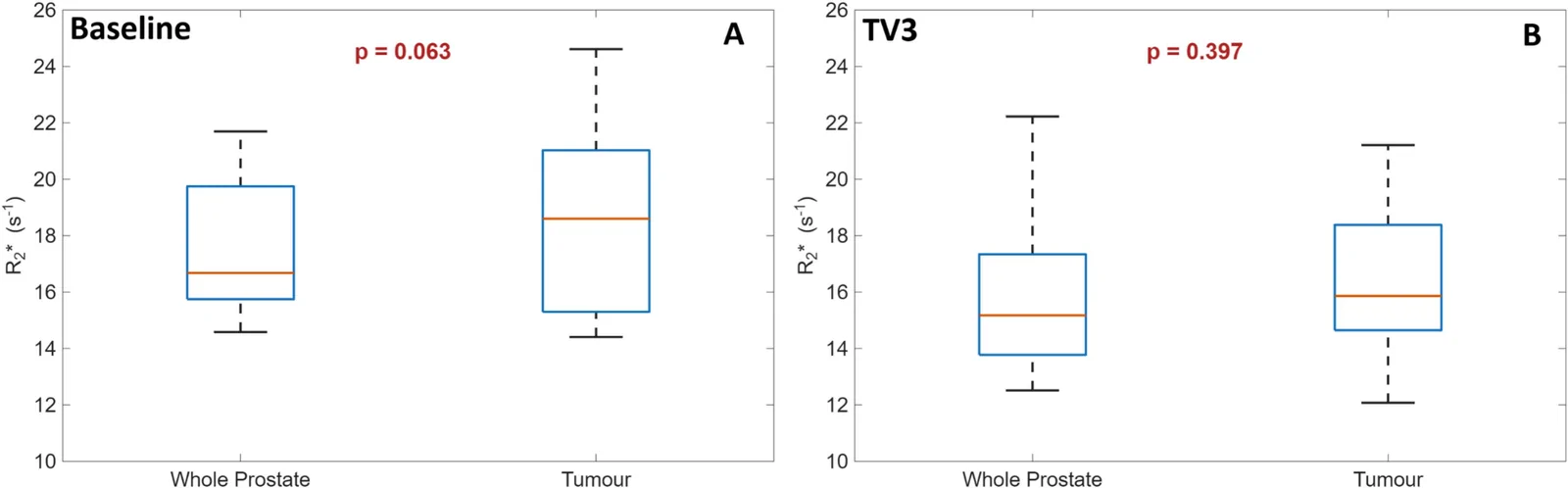
Comparisons between whole-prostate and tumour R_2_* measurements at baseline and TV3.

**Fig. 5:**
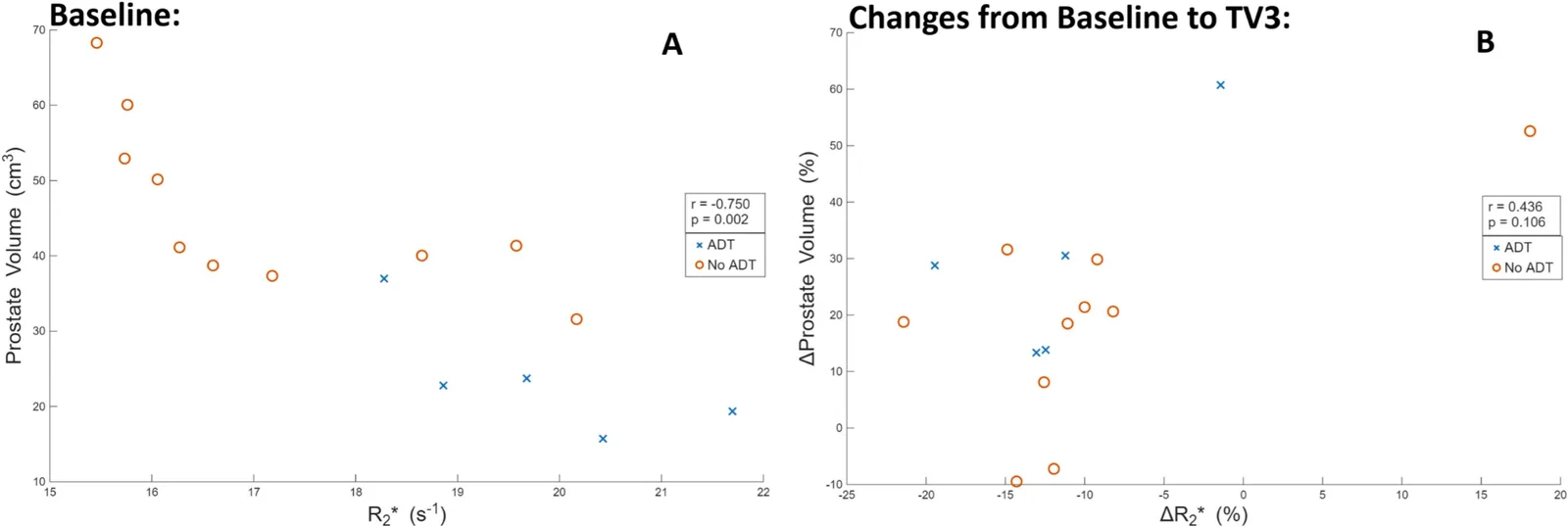
Correlations between whole-prostate R_2_* and volume. Panel A shows the correlation between R_2_* and volume at baseline. Panel B shows the correlation between whole-prostate R_2_* and prostate volume differences between baseline and TV3.

## 4 Discussion

In this feasibility study we have shown it is possible to measure longitudinal R_2_* in the prostate on an MRI-linac in patients treated with five-fraction SABR, with good repeatability measured for whole-prostate R_2_*. Whole-prostate R_2_* showed an acute decrease associated with SABR treatment, observed after two fractions had been delivered. Notably, this change is earlier than in conventional treatment, where an R_2_* decrease was only observed after the conclusion of treatment by Bano et al. [16]. We observed a significant increase in prostate volume, consistent with prostate swelling in response to radiotherapy, accompanying the decrease in R_2_*. This is consistent with previous reports of mid-SABR treatment prostate volume increases measured after 2 or 3 fractions, although we measured greater volume changes [23], [24].

For the patients who received follow-up imaging approximately three months post-treatment, the whole-prostate R_2_* and volume measurements appear to return to levels consistent with their pre-treatment values, suggesting that the measured effect is transient. However, given the low number of patients these results (particularly for R_2_* where the change is small) should be confirmed in a larger patient cohort.

R_2_* measurements could be affected by multiple factors including susceptibility effects e.g. from bowel gas, patient motion during acquisition, contouring or registration errors; however, the strong cohort-level response and the correlation with treatment delivery suggests that there is at least some biological effect occurring.

Decreasing tissue hypoxia could be one of the mechanisms contributing to the decrease in R_2_* [9] because of, or as well as, increased oxygen delivery as result of radiation-induced hyperaemia (increased blood flow) [25]. Another possible cause could be radiation-induced oedema (swelling), as increasing fluid content would decrease R_2_ and thereby decrease R_2_* [25].

The strong correlation between baseline whole-prostate R_2_* and volume, and the effects of ADT (which is known to reduce the volume of the prostate [26]) on baseline R_2_* suggest that prostate size, which may reflect fluid content, is a significant factor for R_2_*. The suggestion that R_2_* and volume return to baseline following the conclusion of treatment, after swelling has subsided, appeared to support this but the number of patients in the follow-up cohort is too low to definitively show this. However, the lack of correlation between treatment-induced prostate volume R_2_* changes suggests that swelling is not the only factor contributing to R_2_* changes (if R_2_* was simply a proxy for prostate size, then we would expect a strong, negative correlation in response to treatment). There was no significant difference between whole-prostate and DIL R_2_* before or after treatment, indicating no detectable tumour-specific response to treatment. This is notable, as it may limit the utility of using R_2_* in biologically-guided radiotherapy if there is no differential baseline measurement or response between tissue types.

This study had several limitations. Due to the constraints of the clinical workflow, it was not possible to acquire R_2_* image at every visit. The number of patients was low (*n* = 15), particularly for the follow-up cohort (*n* = 6), so these findings require further corroboration in larger patient cohorts. There were several factors which added uncertainty to the DIL R_2_* measurements. The image quality of the T_2_-weighted anatomical image was sometimes insufficient to clearly delineate the DIL in all cases, and despite image registration between T_2_ and T_2_* there remained some uncertainty, with small DILs on the prostate boundary particularly sensitive to errors. This is reflected in poor DIL volume repeatability (Fig. 1D). While the patients received enemas before imaging, R_2_* maps were susceptible to artefacts from bowel gas which could obscure the prostate/DIL at longer echo times, potentially resulting in non-tissue related R_2_* decreases. However, image quality review attempted to remove potentially artefactual R_2_* values from analysis to reduce the effect of this source of error.

Further work could investigate clinical outcomes to assess whether changes in R_2_* or prostate volume are associated with patient reported side-effects and evaluate their potential relationship with long-term survival and treatment-related toxicity. For example, in our cohort patients experienced similar R_2_* and prostate volume changes, so it would be instructive to assess if these correspond to similar outcomes and side-effects associated with swelling. In a future cohort, R_2_ mapping could be included to try to separate out the fluid-related effects of swelling, as well as imaging at additional timepoints during and after treatment to determine exactly when the changes occur. The signal-to-noise ratio of the T_2_w images should be improved, and/or additional imaging such as diffusion-weighted imaging (DWI) should be incorporated, to aid the accuracy of the delineation of DILs. Further quantitative imaging techniques could be tested and compared to R_2_* and prostate volume changes to assess whether they were associated with inflammation, or some other cause. For example, DWI may be able to detect acute changes to cellularity to determine if there are any radiation induced changes to the tumour [27], and dynamic contrast-enhanced imaging may be able to detect changes in perfusion to the prostate to help determine if R_2_* changes can be linked to changing blood supply [28]. Currently, doses are distributed approximately uniformly over the prostate, which may explain the apparent uniform treatment response between whole-prostate and DIL. The DESTINATION 2 trial (NCT06638541) is exploring ultra hypo-fractionation, delivering treatments in two fractions with de-escalated dose to healthy prostate, using the MRI-linac to visualise the DIL and adapt daily doses. This may provide the opportunity to investigate whether treatment induced R_2_* effects are indeed dose dependent and result in heterogeneous R_2_* changes throughout the prostate.

## CRediT authorship contribution statement

**Chris Moore:** Writing – review & editing, Writing – original draft, Methodology, Investigation, Formal analysis, Data curation. **Asher Ezekiel:** Formal analysis, Data curation, Conceptualization. **Vodathi Bamunuarachchi:** Data curation. **Ereny Saad:** Data curation. **Anubhav Datta:** Methodology, Data curation. **Peter Hoskin:** Writing – review & editing. **Ananya Choudhury:** Writing – review & editing, Resources, Funding acquisition. **Marcel van Herk:** Writing – review & editing, Supervision. **David L. Buckley:** Writing – review & editing, Supervision. **Michael J. Dubec:** Writing – review & editing, Supervision, Conceptualization. **Damien J. McHugh:** Writing – review & editing, Supervision, Software, Formal analysis, Conceptualization.

## Ethics declaration

**Informed consent and patient details**

Written informed consent to take part in the study and to publish the article has been obtained from all participants or their legal representatives. The privacy rights of participants have been observed.

**Studies in Human**

This study was performed in compliance with relevant laws, regulatory frameworks and guidelines where the research took place. Ethics committee approval was not required under relevant laws and institutional guidelines. Retrospective study.

## Declaration of competing interest

The authors declare that they have no known competing financial interests or personal relationships that could have appeared to influence the work reported in this paper.
